# Predicting *In Vitro* Rumen VFA Production Using CNCPS Carbohydrate Fractions with Multiple Linear Models and Artificial Neural Networks

**DOI:** 10.1371/journal.pone.0116290

**Published:** 2014-12-31

**Authors:** Ruilan Dong, Guangyong Zhao

**Affiliations:** College of Animal Science and Technology, China Agricultural University, State Key Laboratory of Animal Nutrition, Beijing, China; University of Florida, United States of America

## Abstract

The objectives of this trial were to develop multiple linear regression (MLR) models and three-layer Levenberg-Marquardt back propagation (BP3) neural network models using the Cornell Net Carbohydrate and Protein System (CNCPS) carbohydrate fractions as dietary variables for predicting *in vitro* rumen volatile fatty acid (VFA) production and further compare MLR and BP3 models. Two datasets were established for the trial, of which the first dataset containing 45 feed mixtures with concentrate/roughage ratios of 10∶90, 20∶80, 30∶70, 40∶60, and 50∶50 were used for establishing the models and the second dataset containing 10 feed mixtures with the same concentrate/roughage ratios with the first dataset were used for testing the models. The VFA production of feed samples was determined using an *in vitro* incubation technique. The CNCPS carbohydrate fractions (g), i.e. CA (sugars), CB_1_ (starch and pectin), CB_2_ (available cell wall) of feed samples were calculated based on chemical analysis. The performance of MLR models and BP3 models were compared using a paired *t*-test, the determination coefficient (R^2^) and the root mean square prediction error (RMSPE) between observed and predicted values. Statistical analysis indicated that VFA production (mmol) was significantly correlated with CNCPS carbohydrate fractions (g) CA, CB_1_, and CB_2_ in a multiple linear pattern. Compared with MLR models, BP3 models were more accurate in predicting acetate, propionate, and total VFA production while similar in predicting butyrate production. The trial indicated that both MLR and BP3 models were suitable for predicting *in vitro* rumen VFA production of feed mixtures using CNCPS carbohydrate fractions CA, CB_1_, and CB_2_ as input dietary variables while BP3 models showed greater accuracy for prediction.

## Introduction

Microbial fermentation of dietary carbohydrates in rumen is characterized by formation of volatile fatty acids (VFA) and gas (CH_4_, CO_2_, and H_2_) [1]. Of the total VFA produced in rumen, acetate, propionate and butyrate account for more than 95% [2]. Since VFA is utilized as the major energy source by ruminants, predicting VFA production in rumen fermentation is of importance for evaluating the energy supply for ruminants.

The VFA in the rumen is produced in the process of the microbial fermentation of dietary carbohydrates and the amount of VFA produced is mainly depended both upon feed intake and chemical composition, particularly the nature and degradation rate of carbohydrates [3]. Existing models for predicting VFA production in rumen including mechanistic models [4], [5] and empirical regression models using feed composition [6], quadratic models using odd and branched chain fatty acids (OBCFA) in milk [7] and multivariate regression models using OBCFA in milk [8], etc. Selecting suitable modeling methods and input variables for predicting rumen VFA production is important to maximize the predicting accuracy of models.

An artificial neural network (ANN) is a parallel, distributed information processing structure consisting of interconnected processing elements (artificial neurons or nodes) [9]. It could identify complex and flexible nonlinear interrelationships between input variables and output variables through hidden nodes without prior assumption [10]. The use of ANN has gained increasing popularity for applications where relationships between dependent and independent variables are either unknown or very complex compared to a traditional regression approach [11]. The key characteristics of ANN make it highly attractive for studying complex rumen fermentation patterns [12].

The Cornell Net Carbohydrate and Protein System (CNCPS) divided the carbohydrates of feeds for ruminants into four fractions, i.e. CA (sugars), CB_1_ (starch and pectin), CB_2_ (available fibre) and CC (unavailable fibre), respectively [13]. Fractions CA, CB_1_, and CB_2_ can be fermented in rumen at fast, moderate, and slow rate, respectively, whereas fraction CC is not fermentable in rumen. The fractionation of dietary carbohydrates by CNCPS reflected not only the carbohydrate composition but also the fermentative characteristics in the rumen. Dong and Zhao (2013) reported that the CNCPS carbohydrate fractions CA, CB_1_, and CB_2_ were closely correlated to the *in vitro* rumen total gas and methane production in a multiple linear pattern [14]. Since gas and VFA were produced simultaneously when dietary carbohydrates were fermented in the rumen, it could be speculated that CNCPS carbohydrate fractions CA, CB_1_, and CB_2_ could be suitable variables for predicting VFA production.

The objectives of the present trial were to model *in vitro* rumen VFA production using the CNCPS carbohydrate fractions as dietary variables using multiple linear regression (MLR) and ANN and further compare the modeling accuracy between MLR and ANN models.

## Materials and Methods

The project involved 6 phases: (1) Establishment of two datasets, of which the first one containing 45 feed mixtures and the second one containing 10 other feed mixtures; (2) The calculation of the CNCPS carbohydrate fractions CA, CB_1_, and CB_2_ of 55 feed mixtures based on chemical analysis; (3) Determination of the VFA production of 55 feed mixtures using an *in vitro* rumen incubation technique; (4) Development of MLR and BP3 models based on the first dataset; (5) Evaluation of MLR and BP3 models using the second dataset; (6) Comparison of MLR and BP3 models.

### Establishment of datasets

Two datasets were established for the trial. The first one for modeling contained 45 feed mixtures with concentrate/roughage ratios of 10∶90, 20∶80, 30∶70, 40∶60, and 50∶50, of which each ratio contained 9 feed mixtures (Table 1). The second one used for evaluating models contained 10 feed mixtures with the same concentrate/roughage ratios, of which each ratio contained 2 feed mixtures (Table 2). The feed mixtures in the trial were formulated based on the components and concentrate/roughage ratios of the actual typical rations for beef cattle in China.

**Table 1:** The components of feed mixtures for modeling (%, air dry basis).

Feed mixture	Corn	Soybean meal	Wheat bran	Cottonseed meal	Rapeseed meal	DDGS	Wheat middlings	Rice straw	Corn stover	Corn silage	Wheat straw	Millet straw	Chinese wildrye	Concentrate/roughage ratio
1	28.5	11.5	10.0	-	-	-	-	50	-	-	-	-	-	50∶50
2	22.8	9.2	8.0	-	-	-	-	60	-	-	-	-	-	40∶60
3	17.1	6.9	6.0	-	-	-	-	70	-	-	-	-	-	30∶70
4	11.4	4.6	4.0	-	-	-	-	80	-	-	-	-	-	20∶80
5	28.5	11.5	10.0	-	-	-	-	-	-	25	-	25	-	50∶50
6	22.8	9.2	8.0	-	-	-	-	-	-	39	-	21	-	40∶60
7	17.1	6.9	6.0	-	-	-	-	-	-	56	-	14	-	30∶70
8	5.7	2.3	2.0	-	-	-	-	-	-	45	-	45	-	10∶90
9	27.5	-	9.5	13.0	-	-	-	-	50	-	-	-	-	50∶50
10	22.0	-	7.6	10.4	-	-	-	-	60	-	-	-	-	40∶60
11	11.0	-	3.8	5.2	-	-	-	-	80	-	-	-	-	20∶80
12	5.5	-	1.9	2.6	-	-	-	-	90	-	-	-	-	10∶90
13	26.5	7.5	9.5	-	6.5	-	-	-	-	50	-	-	-	50∶50
14	15.9	4.5	5.7	-	3.9	-	-	-	-	70	-	-	-	30∶70
15	10.6	3.0	3.8	-	2.6	-	-	-	-	80	-	-	-	20∶80
16	5.3	1.5	1.9	-	1.3	-	-	-	-	90	-	-	-	10∶90
17	18.8	6.0	6.0	4.0	-	2.4	2.8	-	-	-	-	-	60	40∶60
18	14.1	4.5	4.5	3.0	-	1.8	2.1	-	-	-	-	-	70	30∶70
19	9.4	3.0	3.0	2.0	-	1.2	1.4	-	-	-	-	-	80	20∶80
20	4.7	1.5	1.5	1.0	-	0.6	0.7	-	-	-	-	-	90	10∶90
21	23.5	7.5	7.5	5.0	-	3.0	3.5	-	25	-	25	-	-	50∶50
22	18.8	6.0	6.0	4.0	-	2.4	2.8	-	39	-	21	-	-	40∶60
23	14.1	4.5	4.5	3.0	-	1.8	2.1	-	56	-	14	-	-	30∶70
24	9.4	3.0	3.0	2.0	-	1.2	1.4	-	60	-	20	-	-	20∶80
25	26.0	-	9.0	7.5	7.5	-	-	-	-	-	-	50	-	50∶50
26	20.8	-	7.2	6.0	6.0	-	-	-	-	-	-	60	-	40∶60
27	15.6	-	5.4	4.5	4.5	-	-	-	-	-	-	70	-	30∶70
28	5.2	-	1.8	1.5	1.5	-	-	-	-	-	-	90	-	10∶90
29	26.0	-	9.0	7.5	7.5	-	-	-	-	-	25	25	-	50∶50
30	20.8	-	7.2	6.0	6.0	-	-	-	-	-	39	21	-	40∶60
31	10.4	-	3.6	3.0	3.0	-	-	-	-	-	60	20	-	20∶80
32	5.2	-	1.8	1.5	1.5	-	-	-	-	-	45	45	-	10∶90
33	25.0	7.5	8.5	5.0	4.0	-	-	-	-	-	50	-	-	50∶50
34	15.0	4.5	5.1	3.0	2.4	-	-	-	-	-	70	-	-	30∶70
35	10.0	3.0	3.4	2.0	1.6	-	-	-	-	-	80	-	-	20∶80
36	5.0	1.5	1.7	1.0	0.8	-	-	-	-	-	90	-	-	10∶90
37	20.0	6.0	6.8	4.0	3.2	-	-	-	-	20	30	-	10	40∶60
38	15.0	4.5	5.1	3.0	2.4	-	-	-	-	35	21	-	14	30∶70
39	10.0	3.0	3.4	2.0	1.6	-	-	-	-	52	14	-	14	20∶80
40	5.0	1.5	1.7	1.0	0.8	-	-	-	-	30	30	-	30	10∶90
41	27.0	-	8.0	-	15.0	-	-	-	25	-	25	-	-	50∶50
42	21.6	-	6.4	-	12.0	-	-	-	39	-	21	-	-	40∶60
43	16.2	-	4.8	-	9.0	-	-	-	56	-	14	-	-	30∶70
44	10.8	-	3.2	-	6.0	-	-	-	60	-	20	-	-	20∶80
45	5.4	-	1.6	-	3.0	-	-	-	45	-	45	-	-	10∶90

Note: DDGS refers to dried distiller's grains with solubles.

**Table 2:** The components of feed mixtures for validation (%, air dry basis).

Feed mixture	Corn	Soybean meal	Wheat bran	Cottonseed meal	Rapeseed meal	DDGS	Wheat middlings	Rice straw	Corn stover	Corn silage	Wheat straw	Millet straw	Chinese wildrye	Concentrate/roughage ratio
1	5.7	2.3	2.0	-	-	-	-	90	-	-	-	-	-	10∶90
2	11.4	4.6	4.0	-	-	-	-	-	-	60	-	20	-	20∶80
3	16.5	-	5.7	7.8	-	-	-	-	70	-	-	-	-	30∶70
4	21.2	6.0	7.6	-	5.2	-	-	-	-	60	-	-	-	40∶60
5	23.5	7.5	7.5	5.0	-	3.0	3.5	-	-	-	-	-	50	50∶50
6	4.7	1.5	1.5	1.0	-	0.6	0.7	-	45	-	45	-	-	10∶90
7	10.4	-	3.6	3.0	3.0	-	-	-	-	-	-	80	-	20∶80
8	15.6	-	5.4	4.5	4.5	-	-	-	-	-	56	14	-	30∶70
9	20.0	6.0	6.8	4.0	3.2	-	-	-	-	-	60	-	-	40∶60
10	25.0	7.5	8.5	5.0	4.0	-	-	-	-	30	10	-	10	50∶50

Note: DDGS refers to dried distiller's grains with solubles.

### Trial procedure

Two growing Simmental male cattle, with an average liveweight of 372±6 kg, and fitted with rumen cannulas, were used as the donors of rumen fluid. The daily ration for the cattle consisted of 6.0 kg Chinese wildrye and 2.0 kg concentrate mixture. The concentrate mixture was composed of 58% corn, 20% soybean meal, 18% wheat bran, 2% calcium hydrogen phosphate, 1% sodium chloride, and 1% trace element mixture. The ration was divided into two equal meals and the cattle were fed at 0700 h and 1700 h, respectively. Fresh drinking water was freely available at all times. All procedures involving management of the cattle were according to the approval of the China Agricultural University Institutional Animal Care and Use Committee. We have complied with ethical standards in the treatment of animals. No animals were killed specifically for this trial.

The *in vitro* incubation technique of Menke and Steingass [15] was used for the measurement of acetate, propionate, butyrate, and total VFA production of feed samples. Glass syringes with a calibrated volume of 100 ml were used as the incubation vessels. Two hundred ml of rumen fluid was taken from each cattle through the rumen fistulas 2 h after feeding in the morning. The rumen fluid from the two cattle was well mixed and immediately strained through four layers of gauze into a pre-warmed bottle (39°C). Three hundred ml of rumen fluid and 600 ml buffer were mixed and continuously gassed with carbon dioxide. Each syringe contained 0.2000 g feed sample and the syringes were pre-warmed at 39°C. Four syringes were used for each feed mixture as replicates and three syringes without feed samples were used as the blanks for each batch of samples. Each syringe was filled with 30 ml rumen fluid-buffer mixture. The air in the syringes was transpired and the heads of the syringes were sealed. The syringes were then kept in a water bath at 39°C for incubation for 48 h. At the end of incubation, the pH was immediately measured. An aliquot of 0.80 ml incubation liquid was taken and mixed with 0.20 ml of 25% metaphosphoric acid containing 20 mmol 2-ethyl butyric acid (internal standard). Subsequently the samples were centrifuged at 10, 000× g for 20 min at 4°C to obtain clear supernatant for VFA analysis.

### Chemical analysis

The dry matter (DM), EE, and ash of feed samples were determined according to the methods of no. 934.01, 920.39, and 924.05 of AOAC (1990) [16], respectively. The crude protein (CP) was analyzed using the Kjeldahl method. The neutral detergent fibre (NDF) was analyzed using the method of Van Soest et al. (1991) [17]. The neutral detergent insoluble CP (NDICP) was analyzed by determination of the CP in NDF residues. The acid detergent lignin was analyzed using the method of Goering and Van Soest (1970) [18]. The starch was determined using spectrophotometry (UV-9100, Beijing *Ruili* Analytical Instruments, China) after converting starch to glucose using an enzyme kit containing thermostable α-amylase and amyloglucosidase (Megazyme International Ireland Ltd., Wicklow, Ireland; Method 996.11, AOAC, 1990).

The VFA was analyzed using gas chromatography (TP-2060F, Beijing *Beifen Tianpu* Instrument Technology Co., Ltd., Beijing, China). The conditions for the analysis were as following: FID detector, PEG-20M+H_3_PO_4_ glass capillary column, column temperature 120°C, detector temperature 220°C. The carrying gas was argon, hydrogen and air, with flow rates of 30, 30 and 300 ml/min, respectively. The molar concentrations of acetate, propionate, isobutyrate, butyrate, isovalerate, valerate, and 2-ethyl butyrate (internal standard) in the standard VFA solution were 42, 32.1, 2, 14, 7, 3, and 4 mmol/l, respectively.

### Calculations

The CNCPS carbohydrate fractions of feed mixtures were calculated according to Sniffen et al. [13] and listed in Tables 3 and 4, respectively.
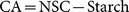


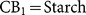


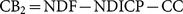


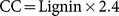


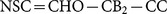


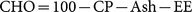
where CA refers to sugars; CB_1_, starch and pectin; CB_2_, available cell wall; CC, unavailable cell wall; NSC, non-structural carbohydrate; CHO, carbohydrate; CP, crude protein; NDICP, neutral detergent insoluble crude protein. The unit for all CNCPS fractions is %DM.

**Table 3:** The CNCPS carbohydrate fractions of feed mixtures for modeling (%DM).

Feed mixture	Carbohydrates	Carbohydrate fractions				NSC
		CA	CB_1_	CB_2_	CC	
1	77.34	10.03	19.56	39.97	7.75	29.60
2	77.95	9.45	15.94	43.96	8.57	25.40
3	78.56	8.87	12.33	47.95	9.40	21.20
4	79.17	8.29	8.71	51.93	10.23	17.00
5	80.68	14.41	19.15	38.13	8.96	33.56
6	82.03	13.99	15.47	43.02	9.51	29.46
7	83.40	13.33	11.81	48.34	9.90	25.14
8	85.78	15.59	4.35	52.61	13.24	19.93
9	76.89	12.74	18.38	36.27	9.48	31.12
10	77.40	13.26	14.82	39.78	9.51	28.08
11	78.41	14.28	7.71	46.82	9.58	21.99
12	78.91	14.80	4.16	50.34	9.62	18.95
13	79.92	12.01	17.92	40.90	9.06	29.93
14	82.93	11.98	11.06	49.86	10.02	23.04
15	84.44	11.96	7.63	54.34	10.49	19.59
16	85.94	11.94	4.21	58.82	10.97	16.15
17	80.35	14.40	13.92	37.91	14.10	28.32
18	81.69	14.57	10.53	41.20	15.38	25.10
19	83.03	14.74	7.14	44.48	16.66	21.88
20	84.37	14.92	3.74	47.77	17.94	18.66
21	77.33	11.78	17.39	35.99	12.15	29.17
22	77.81	12.39	14.03	39.56	11.81	26.41
23	78.11	13.30	10.67	43.14	10.98	23.97
24	79.17	12.88	7.29	46.69	12.29	20.17
25	79.54	15.02	17.36	33.35	13.78	32.38
26	80.97	15.98	13.99	36.51	14.46	29.97
27	82.39	16.95	10.62	39.67	15.13	27.57
28	85.24	18.88	3.88	46.00	16.48	22.76
29	79.19	11.34	17.32	34.48	16.02	28.66
30	80.42	10.24	13.94	38.28	17.94	24.18
31	82.97	9.07	7.17	45.55	21.17	16.24
32	84.61	12.25	3.82	48.03	20.50	16.07
33	78.02	8.92	16.66	35.59	16.83	25.59
34	80.92	7.40	10.15	42.82	20.53	17.55
35	82.37	6.63	6.89	46.44	22.38	13.53
36	83.81	5.87	3.64	50.06	24.24	9.51
37	79.95	10.52	13.48	40.87	15.06	24.00
38	81.75	11.18	10.29	45.82	14.45	21.46
39	83.57	11.57	7.10	51.08	13.81	18.67
40	84.60	10.91	3.75	52.16	17.78	14.66
41	77.32	9.85	17.78	35.44	14.22	27.63
42	77.80	10.84	14.34	39.12	13.47	25.18
43	78.10	12.14	10.91	42.81	12.23	23.04
44	79.16	12.11	7.45	46.47	13.12	19.55
45	81.34	10.14	3.95	50.10	17.14	14.09

**Table 4:** The CNCPS carbohydrate fractions of feed mixtures for evaluating the model (%DM).

Feed mixture	Carbohydrates	Carbohydrate fractions				NSC
		CA	CB_1_	CB_2_	CC	
1	79.78	7.71	5.09	55.92	11.05	12.80
2	84.66	13.71	8.10	51.82	11.02	21.81
3	77.90	13.77	11.27	43.30	9.55	25.04
4	81.42	11.99	14.49	45.38	9.54	26.48
5	79.02	14.22	17.32	34.63	12.82	31.54
6	81.34	10.52	3.87	50.21	16.73	14.39
7	83.82	17.91	7.25	42.84	15.81	25.16
8	81.60	8.70	10.54	42.21	20.14	19.24
9	79.47	8.16	13.41	39.20	18.68	21.57
10	78.73	11.96	16.78	38.21	11.75	28.74

The acetate, propionate, butyrate and total VFA production of feed samples (mmol/g DM of feed mixtures) was calculated as following:
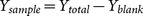
where *Y_sample_* refers to the acetate, propionate, butyrate or total VFA production of feed sample in 48 h; *Y_total_*, the acetate, propionate, butyrate or total VFA production of incubation in 48 h; *Y_blank_*, the acetate, propionate, butyrate or total VFA production of blank in 48 h. The acetate, propionate, butyrate or total VFA production and the pH for modeling were listed in Table 5.

**Table 5:** The acetate, propionate, butyrate, and total VFA production and pH of feed mixtures for modeling.

Feed	Acetate	Propionate	Butyrate	Total VFA	
mixture	(mmol/g DM)	(mmol/g DM)	(mmol/g DM)	(mmol/g DM)	pH
1	1.257±0.023	1.282±0.064	0.772±0.041	3.534±0.131	6.63±0.00
2	1.249±0.088	1.216±0.051	0.653±0.036	3.301±0.178	6.61±0.01
3	1.262±0.021	1.201±0.007	0.653±0.007	3.278±0.023	6.63±0.01
4	1.265±0.057	1.157±0.058	0.532±0.022	3.089±0.128	6.71±0.01
5	1.366±0.023	1.483±0.029	0.783±0.008	3.870±0.035	6.58±0.00
6	1.369±0.020	1.387±0.032	0.722±0.032	3.667±0.086	6.58±0.00
7	1.408±0.020	1.373±0.021	0.615±0.025	3.547±0.045	6.58±0.01
8	1.511±0.065	1.320±0.072	0.500±0.041	3.464±0.177	6.63±0.02
9	1.191±0.019	1.207±0.009	0.725±0.019	3.305±0.019	6.66±0.01
10	1.216±0.027	1.194±0.028	0.695±0.011	3.266±0.067	6.74±0.01
11	1.235±0.020	1.142±0.019	0.588±0.033	3.095±0.076	6.77±0.00
12	1.238±0.042	1.110±0.042	0.447±0.024	2.903±0.098	6.78±0.01
13	1.200±0.025	1.339±0.052	0.651±0.006	3.423±0.031	6.51±0.00
14	1.295±0.097	1.258±0.065	0.588±0.038	3.270±0.198	6.57±0.00
15	1.357±0.042	1.244±0.042	0.524±0.007	3.229±0.082	6.55±0.01
16	1.360±0.035	1.235±0.007	0.387±0.029	3.061±0.073	6.57±0.00
17	1.354±0.030	1.224±0.029	0.747±0.015	3.500±0.059	6.47±0.02
18	1.357±0.018	1.130±0.026	0.655±0.014	3.306±0.052	6.51±0.02
19	1.367±0.025	1.107±0.021	0.569±0.015	3.197±0.048	6.55±0.01
20	1.452±0.084	1.092±0.035	0.499±0.009	3.176±0.113	6.58±0.00
21	1.291±0.067	1.484±0.049	0.753±0.036	3.791±0.115	6.64±0.02
22	1.321±0.070	1.375±0.072	0.640±0.029	3.530±0.174	6.56±0.01
23	1.382±0.029	1.317±0.033	0.584±0.008	3.468±0.077	6.59±0.01
24	1.401±0.039	1.316±0.030	0.571±0.028	3.448±0.099	6.59±0.01
25	1.136±0.020	1.075±0.020	0.782±0.007	3.176±0.035	6.73±0.00
26	1.208±0.043	1.063±0.017	0.699±0.032	3.125±0.085	6.75±0.00
27	1.210±0.092	1.043±0.066	0.606±0.034	2.978±0.183	6.69±0.01
28	1.279±0.043	0.999±0.034	0.414±0.016	2.771±0.095	6.77±0.00
29	1.152±0.048	1.337±0.034	0.624±0.012	3.285±0.060	6.61±0.01
30	1.165±0.047	1.281±0.056	0.610±0.020	3.212±0.105	6.66±0.01
31	1.227±0.035	1.233±0.012	0.527±0.016	3.110±0.033	6.75±0.01
32	1.309±0.060	1.150±0.053	0.483±0.043	3.054±0.090	6.74±0.00
33	0.907±0.036	1.123±0.014	0.682±0.003	2.912±0.029	6.65±0.00
34	0.970±0.026	1.084±0.028	0.551±0.025	2.736±0.063	6.69±0.01
35	0.996±0.034	1.072±0.025	0.530±0.011	2.719±0.074	6.71±0.01
36	1.060±0.050	1.057±0.020	0.366±0.014	2.555±0.030	6.69±0.01
37	1.154±0.031	1.158±0.019	0.611±0.023	3.093±0.067	6.69±0.00
38	1.160±0.008	1.150±0.016	0.601±0.025	3.048±0.020	6.71±0.00
39	1.187±0.018	1.120±0.014	0.509±0.009	2.922±0.030	6.74±0.00
40	1.215±0.080	1.072±0.009	0.445±0.007	2.830±0.072	6.77±0.00
41	1.283±0.055	1.373±0.060	0.616±0.024	3.516±0.103	6.58±0.01
42	1.353±0.027	1.313±0.043	0.577±0.015	3.417±0.089	6.49±0.01
43	1.413±0.005	1.255±0.024	0.555±0.024	3.372±0.042	6.57±0.03
44	1.413±0.082	1.155±0.083	0.485±0.042	3.167±0.211	6.54±0.00
45	1.414±0.048	1.101±0.008	0.362±0.007	2.938±0.054	6.57±0.04

Note: Values were presented as Mean ± Standard error (SE). The unit mmol/g DM refers to mmol VFA per gram DM of feed mixtures.

### Establishment of MLR models

The VFA production and the CNCPS carbohydrate fractions of the first dataset were used to establish the MLR models. Of the carbohydrate fractions, CA, CB_1_, and CB_2_ were used as the effective input parameters for modeling.

The acetate, propionate, butyrate, or total VFA production were used as the dependent variables, and the CNCPS carbohydrate fractions CA, CB_1_ and CB_2_ as the independent variables. The regression relationship between the acetate, propionate, butyrate, or total VFA production (mmol) and the CNCPS carbohydrate fractions (g) was analyzed using the following equation:
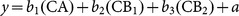
where *y* refers to acetate, propionate, butyrate, or total VFA production; *a*, constant; *b_1_*, *b_2_*, and *b_3_*, coefficients. Since the CNCPS fraction CC is unavailable cell wall in rumen fermentation [19], it was excluded in the equation.

### Establishment of BP3 models

The three layer Levenberg-Marquardt back propagation (BP3) neural network which included an input layer, a hidden layer and an output layer was adopted in the trial. The input layer of network included three input variables, i.e. CA, CB_1_, and CB_2_. The hidden layer containing different number of neurons was a monolayer structure. The output layer included one output variable, i.e. acetate, propionate, butyrate, or total VFA production or four output variables including acetate, propionate, butyrate, and total VFA production simultaneously.

The VFA and the CNCPS carbohydrate fractions of the first dataset were used for training the BP3 neural network on the platform of the MATLAB 7.14 (The Math Works, USA, 2012). The training conditions were: 0.1 for learning rate, 1000 for training epochs, and 0.00001 for goal of training. The performance of different BP3 models with the number of hidden layer neurons ranging from 1 to16 was compared.

### Validation of models

The VFA and the CNCPS carbohydrate fractions of the second dataset were used for validating the MLR models and the BP3 models. The validation was carried out in three ways: Comparing the observed and the predicted VFA values using the *t*-test; analyzing the linear regression relationship between the observed and the predicted VFA values using the model:
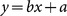
where *x* refers to the observed acetate, propionate, butyrate, or total VFA production, mmol/g DM of feed mixtures; *y* refers to the predicted acetate, propionate, butyrate, or total VFA production, mmol/g DM of feed mixtures; calculating the root mean square prediction error (RMSPE) between the observed and the predicted VFA production:
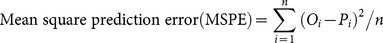


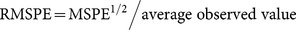
where *i* = 1, 2, …, *n*; *O_i_* refers to the observed value; *P_i_*, the predicted value; *n*, the number of determinations. RMSPE, the ratio of the observed mean used to indicate the whole prediction error, %.

The SAS Statistical Package 9.2 (SAS Institute Inc., Carey, NC, USA) was used for statistical analysis. The MLR relationships between the VFA production and the CNCPS carbohydrate fractions were analyzed using the PROC GLM Procedure. The comparisons between the observed and the predicted values were performed using a *t*-test. The linear relationships between the observed and the predicted values were analyzed using the PROC REG Procedure.

## Results

### *In vitro* incubation

At the end of *in vitro* incubation, the pH value of the incubation residue was within the range of 6.40–6.80, and the microscopic check indicated that the rumen microorganisms were active, indicating the *in vitro* incubation was functioning.

### MLR models

Significant MLR relationships were found between the acetate, butyrate, total VFA, or propionate production (mmol) and the CNCPS carbohydrate fractions CA, CB_1_, and CB_2_ (g).
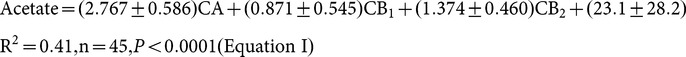


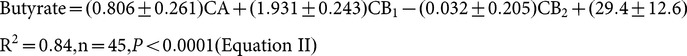


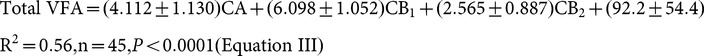


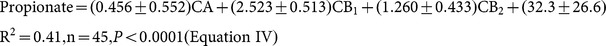



### BP3 models

Relationships between the number of neurons in hidden layer, the R^2^ and the RMSPE values between the observed and the predicted VFA production were presented in Tables 6 and 7, respectively.

**Table 6:** The R^2^ between the observed and the predicted values with different number of nodes in the hidden layer.

Number of Nodes	Acetate	Propionate	Butyrate	Total VFA	Acetate, propionate, butyrate and total VFA together as output variables			
					Acetate	Propionate	Butyrate	Total VFA
1	0.10	0.10	0.933	0.16	0.02	0.02	0.64	0.28
2	0.04	0.11	0.935	0.26	0.58	0.03	0.80	0.38
3	0.47	0.50	0.888	0.25	0.58	0.06	0.82	0.32
4	0.74	0.14	0.856	0.44	0.19	0.24	0.82	0.26
5	0.43	0.28	0.789	0.21	0.53	0.08	0.87	0.27
6	0.74	0.44	0.930	0.57	0.34	0.25	0.92	0.32
7	0.48	0.33	0.907	0.34	0.71	0.39	0.83	0.50
8	0.28	0.15	0.928	0.27	0.52	0.02	0.85	0.26
9	0.39	0.14	0.871	0.38	0.47	0.12	0.81	0.25
10	0.11	0.35	0.891	0.29	0.62	0.31	0.79	0.58
11	0.70	0.24	0.838	0.47	0.28	0.32	0.86	0.15
12	0.19	0.05	0.921	0.19	0.50	0.32	0.90	0.51
13	0.65	0.28	0.783	0.43	0.49	0.19	0.87	0.31
14	0.05	0.16	0.937	0.44	0.17	0.18	0.66	0.06
15	0.23	0.14	0.341	0.54	0.31	0.24	0.82	0.42
16	0.07	0.30	0.920	0.49	0.40	0.39	0.69	0.43

**Table 7:** The RMSPE (%) between the observed and the predicted values with different number of nodes in the hidden layer.

Number of Nodes	Acetate	Propionate	Butyrate	Total VFA	Acetate, propionate, butyrate and total VFA together as output variables			
					Acetate	Propionate	Butyrate	Total VFA
1	11.25	7.89	4.38	7.07	11.19	8.72	9.45	6.81
2	13.48	8.85	4.49	6.79	7.44	10.33	10.60	6.97
3	8.12	5.75	7.40	7.00	7.37	8.13	7.77	6.66
4	5.73	8.31	7.16	7.10	10.05	7.83	7.46	7.15
5	8.68	7.86	8.27	11.43	7.95	8.74	7.73	7.93
6	5.77	8.39	4.24	5.46	9.10	7.63	5.01	7.02
7	8.14	9.65	5.68	7.70	6.14	7.59	8.25	6.12
8	17.04	9.21	6.45	6.95	7.93	10.10	8.48	8.89
9	12.83	8.21	9.55	7.74	8.15	8.55	9.22	8.01
10	12.72	7.22	5.32	11.08	7.86	8.18	8.49	6.39
11	7.00	11.25	12.27	5.72	9.51	9.80	12.65	8.62
12	10.77	14.59	4.96	8.32	8.03	7.46	7.20	5.61
13	6.71	10.00	13.05	8.06	8.66	8.22	8.33	7.96
14	17.98	11.12	8.16	8.84	11.71	9.97	10.01	14.62
15	13.16	14.06	21.88	10.25	11.72	8.45	7.69	7.67
16	16.91	10.25	6.51	8.50	10.96	7.30	10.49	9.33

**Figure 1:**
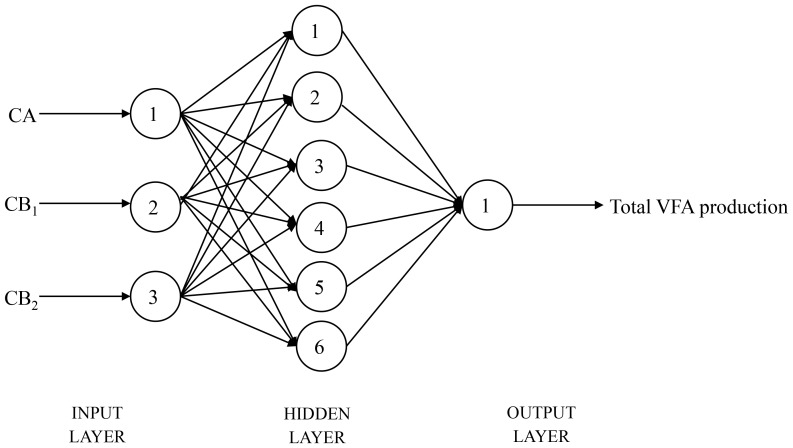
The basic structure of back propagation neural network for total VFA production.

**Figure 2:**
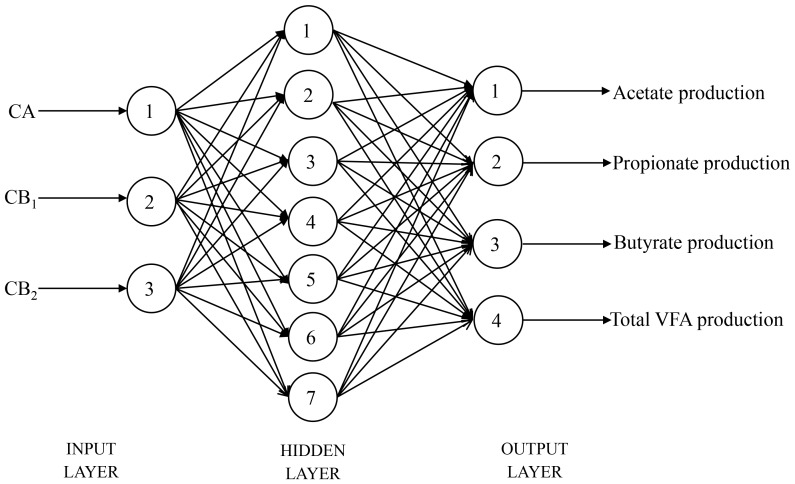
The basic structure of back propagation neural network for acetate, propionate, butyrate, or total VFA production output simultaneously.

By training for 1000 times, the target error was converged to 0.00001 and then training was ended. The best structures of the BP3 for predicting acetate, propionate, butyrate and total VFA production were 3—4—1, 3—3—1, 3—6—1 and 3—6—1 (Fig. 1), respectively. The best structure of the BP3 established for predicting acetate, propionate, butyrate and total VFA production simultaneously was 3—7—4 (Fig. 2).

### Evaluation of MLR and BP3 models

Results of the observed *versus* MLR and BP3 predicted values for individual or simultaneous output of acetate, propionate, butyrate, and total VFA production using evaluation rations are presented in Tables 8 and 9, respectively. Paired *t*-test showed that no differences were found between the observed and the predicted acetate, butyrate, total VFA, and propionate production based on Equations I (*P* = 0.817), II (*P* = 0.324), III (*P* = 0.965) and IV (*P* = 0.981), respectively.

**Table 8:** Comparison of observed and predicted acetate, propionate, butyrate, and total VFA production using evaluation rations.

Feed mixture	Acetate (mmol/g DM)			Propionate (mmol/g DM)			Butyrate (mmol/g DM)			Total VFA (mmol/g DM)			pH
	Observed	MLR predicted	BP3 predicted	Observed	MLR predicted	BP3 predicted	Observed	MLR predicted	BP3 predicted	Observed	MLR predicted	BP3 predicted	
1	1.327±0.069	1.257	1.333	1.157±0.032	1.192	1.197	0.467±0.026	0.437	0.485	3.071±0.093	2.983	3.109	6.69±0.01
2	1.435±0.026	1.393	1.325	1.337±0.032	1.243	1.268	0.570±0.036	0.544	0.548	3.486±0.059	3.308	3.411	6.62±0.01
3	1.227±0.026	1.305	1.295	1.147±0.014	1.216	1.217	0.644±0.015	0.609	0.608	3.169±0.060	3.286	3.478	6.76±0.01
4	1.289±0.077	1.312	1.290	1.317±0.029	1.316	1.279	0.646±0.015	0.656	0.651	3.405±0.106	3.462	3.422	6.50±0.01
5	1.329±0.058	1.251	1.273	1.275±0.046	1.262	1.218	0.764±0.028	0.732	0.718	3.563±0.134	3.451	3.414	6.46±0.01
6	1.409±0.088	1.245	1.335	1.298±0.033	1.102	1.168	0.461±0.033	0.437	0.449	3.287±0.142	2.878	2.965	6.65±0.01
7	1.253±0.039	1.378	1.319	1.031±0.032	1.128	1.042	0.556±0.017	0.565	0.550	2.941±0.071	3.199	2.901	6.78±0.01
8	1.203±0.032	1.143	1.139	1.260±0.047	1.161	1.189	0.556±0.022	0.554	0.546	3.158±0.102	3.005	2.963	6.71±0.01
9	0.923±0.080	1.112	0.981	1.102±0.049	1.193	1.192	0.564±0.022	0.606	0.607	2.738±0.147	3.080	2.772	6.71±0.01
10	1.152±0.030	1.233	1.273	1.180±0.018	1.283	1.229	0.697±0.011	0.702	0.703	3.218±0.062	3.417	3.410	6.62±0.01

Note: Values were presented as Mean ± Standard error (SE). The unit mmol/g DM refers to mmol VFA per gram DM of feed mixtures.

The MLR predicted value for acetate, butyrate, total VFA, and propionate production respectively, was calculated by using Equation I, II, III, and IV respectively. The BP3 predicted value for acetate, propionate, butyrate, and total VFA production was individual modeled output respectively using BP3 models.

**Table 9:** Comparison of observed and predicted acetate, propionate, butyrate, and total VFA production using evaluation rations.

Feed mixture	Acetate (mmol/g DM)			Propionate (mmol/g DM)			Butyrate (mmol/g DM)			Total VFA (mmol/g DM)			pH
	Observed	MLR predicted	BP3 predicted	Observed	MLR predicted	BP3 predicted	Observed	MLR predicted	BP3 predicted	Observed	MLR predicted	BP3 predicted	
1	1.327±0.069	1.257	1.286	1.157±0.032	1.192	1.056	0.467±0.026	0.437	0.363	3.071±0.093	2.983	2.788	6.69±0.01
2	1.435±0.026	1.393	1.363	1.337±0.032	1.243	1.257	0.570±0.036	0.544	0.564	3.486±0.059	3.308	3.322	6.62±0.01
3	1.227±0.026	1.305	1.316	1.147±0.014	1.216	1.206	0.644±0.015	0.609	0.599	3.169±0.060	3.286	3.272	6.76±0.01
4	1.289±0.077	1.312	1.327	1.317±0.029	1.316	1.287	0.646±0.015	0.656	0.628	3.405±0.106	3.462	3.407	6.50±0.01
5	1.329±0.058	1.251	1.217	1.275±0.046	1.262	1.199	0.764±0.028	0.732	0.739	3.563±0.134	3.451	3.352	6.46±0.01
6	1.409±0.088	1.245	1.307	1.298±0.033	1.102	1.121	0.461±0.033	0.437	0.460	3.287±0.142	2.878	2.997	6.65±0.01
7	1.253±0.039	1.378	1.218	1.031±0.032	1.128	0.968	0.556±0.017	0.565	0.515	2.941±0.071	3.199	2.819	6.78±0.01
8	1.203±0.032	1.143	1.108	1.260±0.047	1.161	1.179	0.556±0.022	0.554	0.594	3.158±0.102	3.005	3.044	6.71±0.01
9	0.923±0.080	1.112	0.951	1.102±0.049	1.193	1.169	0.564±0.022	0.606	0.614	2.738±0.147	3.080	2.905	6.71±0.01
10	1.152±0.030	1.233	1.248	1.180±0.018	1.283	1.287	0.697±0.011	0.702	0.762	3.218±0.062	3.417	3.506	6.62±0.01

Note: Values were presented as Mean ± Standard error (SE). The unit mmol/g DM refers to mmol VFA per gram DM of feed mixtures.

The MLR predicted value for acetate, butyrate, total VFA, and propionate production respectively, was calculated by using Equation I, II, III, and IV respectively. The BP3 predicted value for acetate, propionate, butyrate, and total VFA production was modeled output simultaneously using BP3 architecture 3—7—4 with 7 neurons in the hidden layer.

**Figure 3:**
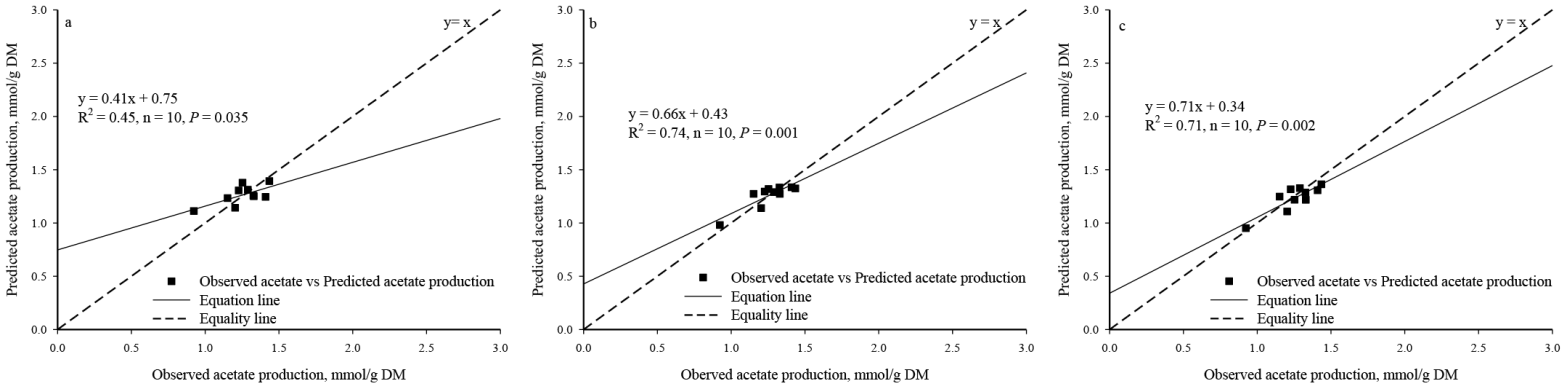
Relationship between the observed *vs.* the predicted acetate production. (*a*. Predicted values were from Equation I; *b*. Predicted values were from BP3 model where acetate production using as individual output; *c*. Predicted values were from BP3 model where acetate production was output simultaneously.)

**Figure 4:**
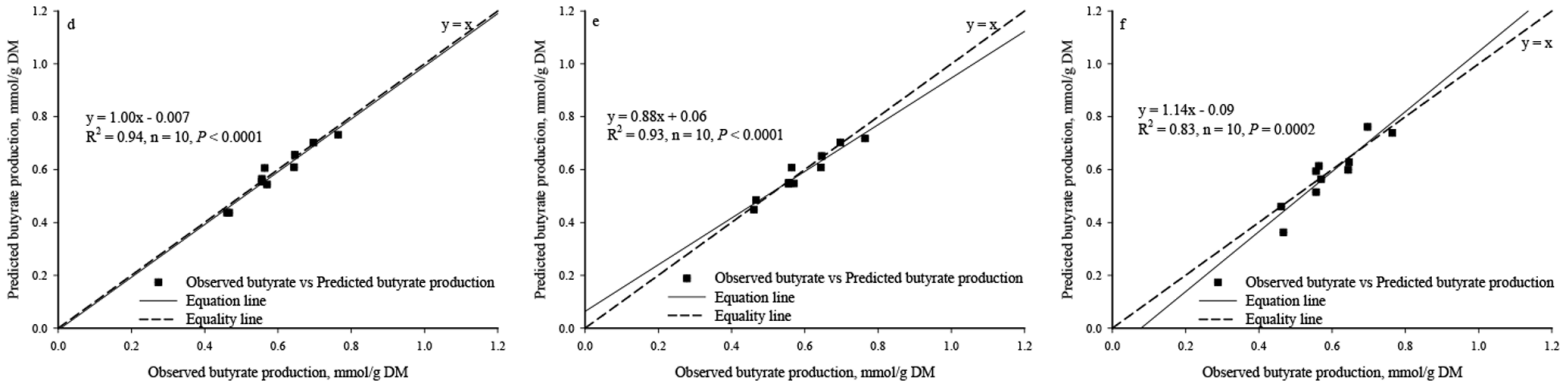
Relationship between the observed *vs.* the predicted butyrate production. (*d*. Predicted values were from Equation II; *e*. Predicted values were from BP3 model where butyrate production using as individual output; *f*. Predicted values were from BP3 model where butyrate production was output simultaneously.)

**Figure 5:**
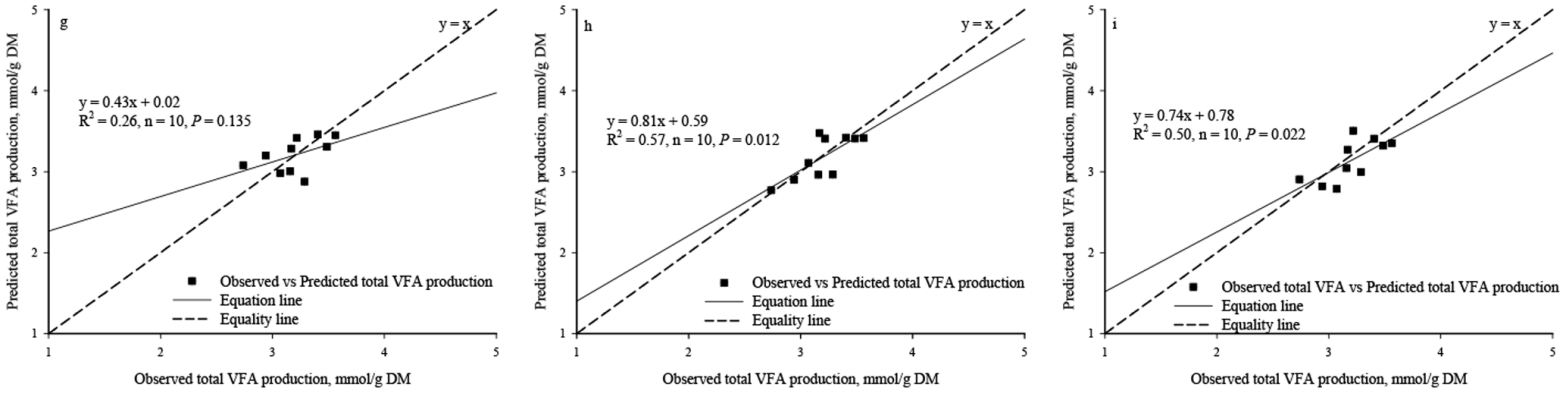
Relationship between the observed *vs.* the predicted total VFA production. (*g*. Predicted values were from Equation III; *h*. Predicted values were from BP3 model where total VFA production using as individual output; *i*. Predicted values were from BP3 model where total VFA production was output simultaneously.)

**Figure 6:**
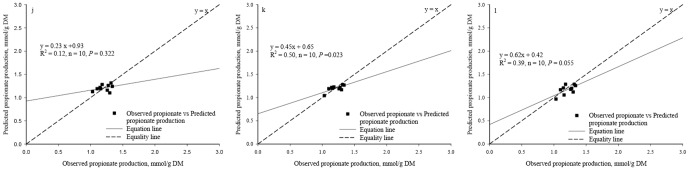
Relationship between the observed *vs.* the predicted propionate production. (*j*. Predicted values were from Equation IV; *k*. Predicted values were from BP3 model where propionate production using as individual output; *l*. Predicted values were from BP3 model where propionate production was output simultaneously.)

Significant regression relationships were found between the observed and the predicted acetate production (R^2^ = 0.45, *P* = 0.035, n = 10, Fig. 3*a*) and butyrate production (R^2^ = 0.94, *P*<0.0001, n = 10, Fig. 4*d*) whereas no significant regression relationships were found between the observed and the predicted total VFA production (R^2^ = 0.26, *P* = 0.135, n = 10, Fig. 5*g*) and the propionate production (R^2^ = 0.12, *P* = 0.322, n = 10, Fig. 6*j*).

No differences were found between the observed and the predicted acetate, propionate, butyrate and total VFA production based on the individual output BP3 models (*P* = 0.948, 0.658, 0.483 and 0.749, respectively) or between the observed and the predicted acetate, propionate, butyrate and total VFA production based on the simultaneous output BP3 model (*P* = 0.427, 0.213, 0.599 and 0.341, respectively).

Based on the single output BP3 models, a significant regression relationship was found between the observed and the predicted values in the acetate production (R^2^ = 0.74, *P* = 0.001, n = 10, Fig. 3*b*), the butyrate production (R^2^ = 0.93, *P*<0.0001, n = 10, Fig. 4*e*), the predicted total VFA production (R^2^ = 0.57, *P* = 0.012, n = 10, Fig. 5*h*), and the propionate production (R^2^ = 0.50, *P* = 0.023, n = 10, Fig. 6*k*).

Based on the simultaneous output BP3 model, a significant regression relationship was found between the observed and the predicted values in the acetate production (R^2^ = 0.71, *P* = 0.002, n = 10, Fig. 3*c*), the butyrate production (R^2^ = 0.83, *P* = 0.0002, n = 10, Fig. 4*f*) and the total VFA production (R^2^ = 0.50, *P* = 0.022, n = 10, Fig. 5*i*). Relationship between the observed and the predicted propionate production tended to be significant (R^2^ = 0.39, *P* = 0.055, n = 10, Fig. 6*l*).

The values of RMSPE of Equations I, II, III and IV were 8.26%, 4.26%, 6.85% and 7.91%, respectively. Based on the individual output BP3 models, the values of RMSPE were 5.73%, 5.75%, 4.24% and 5.46% for acetate, propionate, butyrate, and total VFA production, respectively. Based on the simultaneous output BP3 model, the values of RMSPE were 6.14%, 7.59%, 8.25% and 6.12% for acetate, propionate, butyrate, and total VFA production, respectively.

## Discussion

### *In vitro* incubation

The *in vitro* gas production technique provides a reflection of amounts and proportions of VFA. Because of close correlation between the stoichiometry of short chain fatty acid (SCFA) within the rumen and the *in vitro* gas production [20], *in vitro* gas production technique can be used as a dynamic estimate of feed fermentation rate to predict the rumen fermentation pattern [21]. Hence, the *in vitro* gas production technique could be used as a tool to predict rumen VFA production of feeds.

In the present trial, the pH values of the incubation residues were within the normal range of rumen pH and rumen microorganisms were active at the end of incubation, indicating that using the gas test of Menke and Steingass (1988) was successful. Since the highest predictive value for *in vivo* digestibility of feed was obtained within 45 to 52 h of *in vitro* incubation [22], [23], the VFA production of feed mixtures incubated for 48 h was believed to be reliable.

### Relationships between acetate, propionate, butyrate and total VFA production and CNCPS carbohydrate fractions

Dong and Zhao (2013) reported that the CNCPS carbohydrate fractions CA, CB_1_, and CB_2_ were closely correlated to the *in vitro* rumen total gas and methane production in a multiple linear model and concluded that the CNCPS fractions CA, CB_1_, and CB_2_ were suitable input variables for predicting *in vitro* rumen gas production [14]. Since gas and VFA were produced simultaneously when dietary carbohydrates were fermented in the rumen, it could be presumed that CNCPS CA, CB_1_, and CB_2_ could also be suitable variables for predicting VFA production. Indeed, significant positive regression relationships were found in the present trial between acetate (Equation I), butyrate (Equation II), total VFA production (Equation III), or propionate (Equation IV). The results indicated that the CNCPS carbohydrate fractions CA, CB_1_, and CB_2_ were suitable dietary variables for predicting VFA production. The results were also in accordance with Pitt et al. (1996) who predicted the rate of ruminal VFA production using CA, CB_1_ and CB_2_ carbohydrate fractions [24].

### Effects of input variables, number of neurons and output variables in BP3 models on prediction accuracy

Factors affecting the function of BP3 models include input variables, number of neurons in hidden layer and output variables. More informative or theoretically important parameters used as the input variables were proved to optimize the BP3 model [25]–[27]. In the present trial, the CNCPS carbohydrate fractions CA, CB_1_, and CB_2_ were closely correlated to rumen VFA production in a multiple linear way, hence, CA, CB_1_, and CB_2_ were believed to be suitable input variables of the BP3 models.

When the number of neurons in hidden layer varied from 1 to 16 and acetate, propionate, butyrate, or total VFA production was used as the single output variable, the values of R^2^ varied within the ranges of 0.04–0.74, 0.05–0.50, 0.341–0.937, and 0.16–0.57 and the values of RMSPE varied within the ranges of 5.73–17.98%, 5.75–14.59%, 4.24–21.88%, and 5.46–11.43% for acetate, propionate, butyrate, and total VFA production, respectively, whereas when acetate, propionate, butyrate and total VFA production were used as simultaneous output variables, the values of R^2^ varied within the ranges of 0.02–0.71, 0.02–0.39, 0.64–0.92, and 0.06–0.58 and the values of RMSPE varied within the ranges of 6.14–11.72%, 7.30–10.33%, 5.01–12.65%, and 5.61–14.62%, respectively. The results indicated that the number of neurons in the hidden layer as well as the number of variables in output layer affected the performance of the BP3 models for predicting the acetate, propionate, butyrate, and total VFA production. The variation of R^2^ and RMSPE showed irregularity and the reason was unclear. By comparison of the values of R^2^ and RMSPE of different BP3 models, the BP3 models 3—4—1, 3—3—1, 3—6—1 and 3—6—1 yielded maximum R^2^ and minimum RMSPE for predicting acetate, propionate, butyrate and total VFA production, respectively, and were considered to be most suitable BP3 models for predicting VFA production in the present trial.

### Comparison between MLR and BP3 models

MLR modeling is an ideal tool for identifying linear relationships between different parameters and therefore is widely used in animal science. However, MLR modeling suffers from the prior assumption that the relationships between different parameters follow the assumptions underlying MLR. The advantages of BP3 over MLR modeling include that it could model nonlinear and complex relationships between inputs and outputs and it does not need priori assumption [28]. Therefore, BP3 could be more suitable than MLR for modeling the complex physiological processes in animals [29].

Some studies indicated that ANN modeling was an approach that refined the accuracy of predicting models and showed better predicting performance in sheep [30], [31], goats [32], [33] and dairy cows [34]–[37]. Many studies indicated that ANN models showed better performance than MLR models. The ANN models gave better estimates of total milk production [35], dairy manure nutrient content and layer manure composition [38], [39], the first lactation 305-day milk yield in Kenyan Holstein-Friesian dairy cows [40], body weight in goats and feed abrasive value [41] than MLR models. The results in the present trial indicated that the BP3 models showed better performance for predicting VFA production than MLR models in greater R^2^ and lower RMSPE even though the performance of BP3 was not significant in predicting butyrate production.

It should be noted that both MLR and BP3 models in the present trial were developed based on the *in vitro* measurement of VFA production. The models need to be validated using *in vivo* trials for predicting acetate, propionate, butyrate, and total VFA production from rumen fermentation of cattle.

## Conclusions

The results of the trial demonstrated that the CNCPS carbohydrate fractions CA, CB_1_ and CB_2_ were suitable dietary variables for predicting rumen VFA production. Both MLR and BP3 models established in the trial can be used to predict *in vitro* rumen VFA production of feed mixtures for ruminants while BP3 models performed better in accuracy than MLR models.
