# Correction: Predicting *In Vitro* Rumen VFA Production Using CNCPS Carbohydrate Fractions with Multiple Linear Models and Artificial Neural Networks

**DOI:** 10.1371/journal.pone.0119740

**Published:** 2015-03-16

**Authors:** The PLOS ONE Staff

In the Funding section, the grant number from the funder the National Natural Science Foundation of China is listed incorrectly. The correct grant number is: 31072055. The complete, correct funding information is as follows: This work was financially supported by the National Natural Science Foundation of China (Project no. 31072055) and The Ministry of Science and Technology of China (Project no. 2011BAD26B03-4). The funders had no role in study design, data collection and analysis, decision to publish, or preparation of the manuscript.
